# Preventing thrombotic events in a case of postpartum ovarian artery aneurysm rupture: clinical challenges and management approaches

**DOI:** 10.1093/jscr/rjad282

**Published:** 2023-05-25

**Authors:** Sina Rasti, Elaheh Zarean, Mohammad S Jafarpisheh, Amir Aria

**Affiliations:** Department of Radiology, School of Medicine, Isfahan University of Medical Science, Isfahan, Iran; Department of Obstetrics and Gynecology, School of Medicine, Isfahan University of Medical Science, Isfahan, Iran; Department of Radiology, School of Medicine, Isfahan University of Medical Science, Isfahan, Iran; Department of Internal Medicine, Alzahra Hospital, Isfahan University of Medical Sciences, Isfahan, Iran

**Keywords:** Aneurysm, Retroperitoneal space, Spontaneous rupture, Venous thromboembolism, Pulmonary embolism

## Abstract

Ovarian artery aneurysm is a rare asymptomatic condition usually diagnosed when it ruptures. It causes massive bleeding, often in the peripartum period of multiparous women, who are already at an increased risk for thromboembolic events. Balancing the bleeding risk against the thrombotic complications remains unexplored in such cases. A 35-year-old woman presented with hemorrhagic shock 3 days after delivering her seventh healthy child. During the emergent exploratory laparotomy, she responded well to the blood transfusion; the stable retroperitoneal hematoma indicated no need to explore it. A subsequent episode of hemodynamic instability necessitated another laparotomy, during which the hematoma was evacuated and both ovarian arteries were ligated. Shortly thereafter, the patient suffered a pulmonary embolism (PE). In multiparous patients presenting with peripartum retroperitoneal hematoma and hemorrhagic shock, exploring the hematoma and ligating the ovarian and uterine arteries may reduce the risk of PE or the need for reoperation.

## INTRODUCTION

Ovarian artery rupture is a rare but potentially life-threatening complication that may occur during pregnancy or shortly after delivery. It can lead to a retroperitoneal hematoma, causing significant maternal morbidity and mortality [1].

Pulmonary embolism (PE) is a well-established complication of both puerperium and hospitalization [2], which can further complicate the management of a retroperitoneal hematoma.

In this report, we present a case of a pregnant woman who suffered ovarian artery rupture and retroperitoneal hematoma following delivery. The patient’s management was complicated by PE, which posed a significant challenge for the physicians involved in her care. We discuss the clinical challenge behind her management and explore the measures that could have been taken to prevent complications in this scenario.

## CASE DESCRIPTION

During a night shift, a 35-year-old gravida 9 para 7 woman was admitted to the emergency department of our tertiary care center due to experiencing dizziness, weakness, and pain on the right side of her abdomen. She had a normal vaginal home-delivery 3 days before her referral, resulting in her seventh healthy term child. She had not been under medical supervision during her pregnancy or delivery. The patient’s family reported no previous underlying medical condition, previous surgery, recent trauma, history of complications during or after her prior or current pregnancy, or drug history. However, they had no information regarding the gestational age or reason behind the two reported previous abortions.

On admission, the patient presented with pallor, delirium, mild bloody vaginal discharges, a swollen tender abdomen, systolic/diastolic blood pressure of 85/40 mmHg, pulse rate (PR) of 96 per minute, temperature of 36.6°C, respiratory rate of 19 per minute and blood oxygen saturation of 95% in room air. She was given ringer serum, fresh frozen plasma, packed red blood cells and platelets. Her baseline hemoglobin (Hb), platelet count, activated partial thromboplastin time and prothrombin time international normalized ratio were 4.2 mg/dL, 434 000 per microliter, 25 s and 1.1, respectively. A bedside abdominal ultrasonography revealed a mixed echo retroperitoneal region in the pelvis, extending up to the right upper quadrant, with a volume of ⁓1300 cm^3^ compatible with a massive hematoma.

A laparotomy was necessary due to the patient’s unstable vital signs and paraclinical findings. During the exploration, all abdominal and pelvic organs were intact, and the hematoma was not expansive or pulsatile. Moreover, the patient’s vital signs and Hb improved after the infusion of serum and blood products during surgery. Therefore, without manipulating the hematoma, the operation ended and the sedated patient was sent to the intensive care unit.

**Figure 1:**
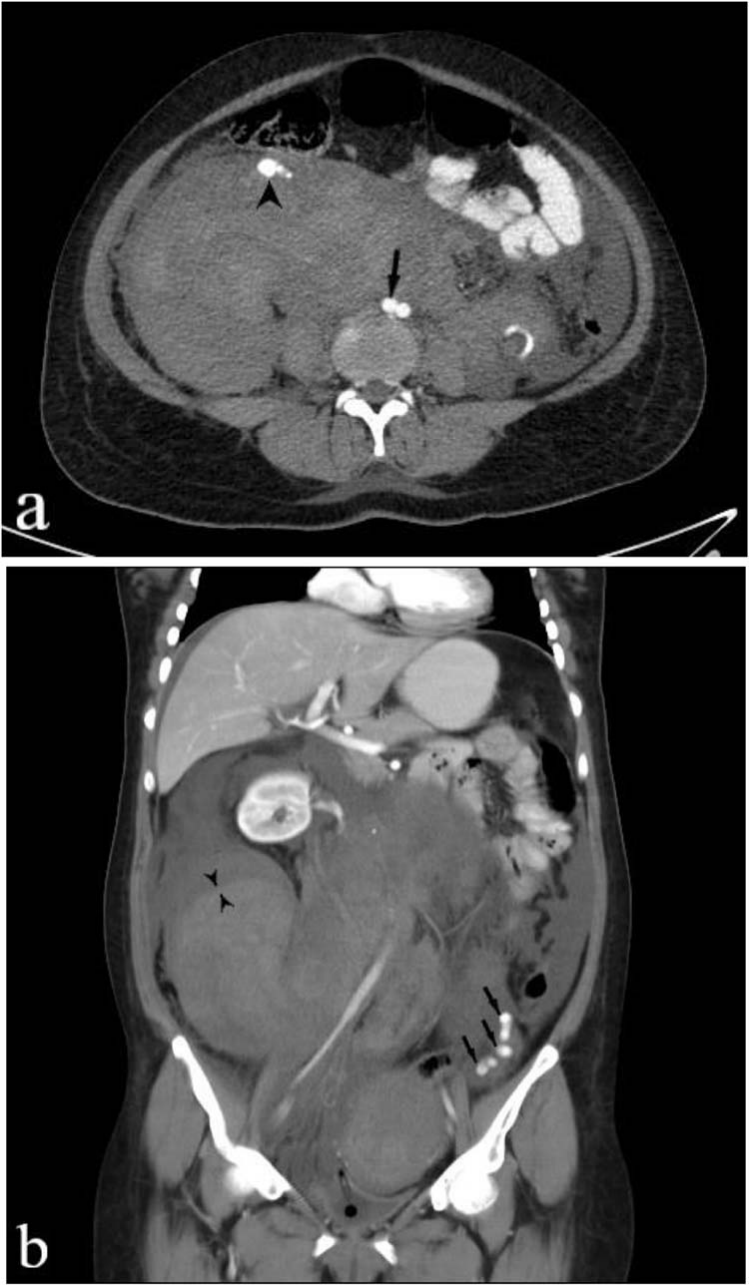
IV and PO contrast-enhanced CT in portal phase shows a massive heterogeneous hematoma in the pelvic region, dominantly in the right lower quadrant. **(a)** An axial section at the level of abdominal aortic bifurcation, shows the dilated and anteriorly displaced right ovarian artery (arrowhead), indicating an aneurysm—its size is comparable to the common iliac artery (arrow). **(b)** A coronal reconstruction of the retroperitoneum demonstrates the extent of hematoma. The left ovarian artery manifests multiple aneurysms (arrows), and the obvious border between two regions of hematoma with different densities (paired arrowheads) shows separate bleeding episodes.

A subsequent emergent abdominopelvic computed tomography (CT) scan with intravenous (IV) and per os (PO) (via nasogastric tube) contrast administration was acquired, disclosing a large heterogeneous retroperitoneal pelvic hematoma extending up around the kidneys, anteriorly displacing the right kidney. Dilated tortuous ovarian arteries were also noticed, with the right-side artery surrounded by hematoma and pushed forward (Fig. 1). These findings indicated ovarian artery aneurysms (OAAs) and the right-side rupture due to delivery as the underlying cause of the massive hemorrhage.

As the heterogeneous hemorrhage demonstrated a constant bleeding state and the right OAA was the most probable source, an urgent transarterial angioembolization (TAE) of the ovarian arteries was planned for the morning. However, the patient’s PR gradually increased, reaching 170 per minute in the morning, despite receiving serum and blood products continuously. Hence, she underwent a second emergent laparotomy. During this procedure, the hematoma was evacuated and active bleeding from the right ovarian artery was noted approximately 2 centimeters distal to the artery's origination from aorta. Ovarian and uterine arteries were ligated bilaterally, and a central venous (CV) catheter was deployed through the right jugular vein for measuring CV pressure and efficient fluid therapy.

**Figure 2:**
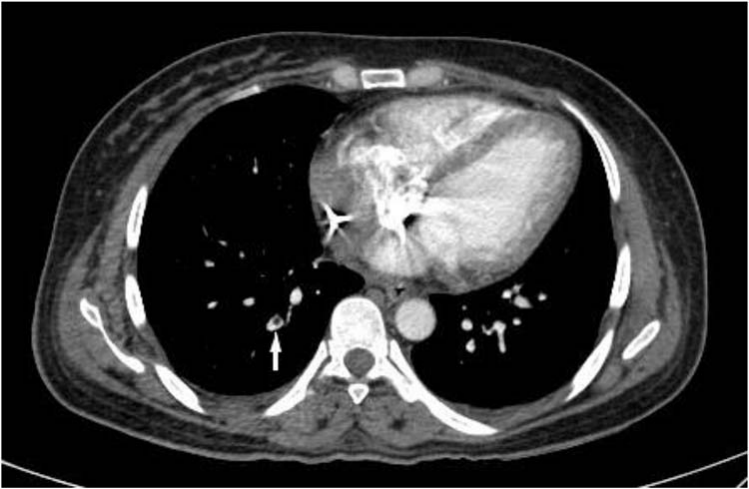
CT pulmonary angiogram on the third day of hospital admission shows a filling defect in a subsegmental branch of the right posterior pulmonary artery (white arrow), compatible with a PE.

Although PRs decreased gradually after the second operation, they increased again after 24 h. This was accompanied by a body temperature rise to 38.7°C. A CT pulmonary angiography (CTPA) was performed following the fever work-up, revealing lobar, segmental and subsegmental filling defects in the posterior branches of the right pulmonary artery (Fig. 2). After confirming the diagnosis of PE, the patient received anticoagulation therapy with caution. Ultimately, without experiencing further complications, the patient survived and was discharged after 3 weeks in stable condition and partial recovery.

Due to the massive hematoma, the patient had not received prophylactic anticoagulation therapy before PE diagnosis. Her only prophylaxis against venous thrombosis was compressive lower limb bandages. During her hospital stay, she showed no evidence of deep venous thrombosis in her lower limbs, so deploying an inferior vena cava filter was never an option for her.

## DISCUSSION

We described a female patient who suffered hemorrhagic shock due to spontaneous retroperitoneal bleeding after delivery. During the emergent laparotomy after admission, surgeons observed a stable hematoma, so they decided not to manipulate it. The subsequent CT scan pictured multiple OAAs surrounded by heterogeneous blood, making her a candidate for bilateral ovarian artery TAE. However, the patient’s hemodynamics deteriorated again, necessitating a second laparotomy. During the second operation the hematoma was evacuated and the ovarian arteries were ligated. Shortly afterwards, the patient showed PE symptoms, confirmed by CTPA.

While OAA is an asymptomatic condition usually found as an incidental finding in imaging studies, its rupture is a life-threatening situation due to massive hemorrhage. Trauma is a potential factor for such a rupture; however, spontaneous ruptures are more difficult to diagnose and the patient tends to lose more blood until diagnosis and treatment. [3] Cases diagnosed with spontaneous ruptured OAA are reported sparsely in the literature probably because of its rarity. These patients are typically multiparous women in their peripartum period of their last pregnancy, just like our case [4]. Transarterial embolization of ovarian arteries has been promising in controlling the condition; nevertheless, its applicability to hemodynamically unstable patients should be further investigated [5, 6].

The most challenging part of managing our patient was her initial surgery. The patient was hemodynamically unstable on arrival but became stable during exploratory laparotomy. This demonstrated an adequate response to aggressive therapy with serum and blood derivatives. Her hematoma was non-pulsatile and non-expansive, and her internal organs were intact. Therefore, the standard of care consisted of preserving the abdominal hematoma in order to tamponade the bleeding [7, 8]. Nevertheless, this management resulted in two complications: the need for a subsequent emergent laparotomy and PE.

To the extent of our knowledge, this is the first report of a peripartum OAA rupture case complicated with PE. However, PE should not be an unexpected condition in such patients legitimately. Our case had several risk factors for developing venous thromboembolism: puerperal state, surgery, massive blood loss, immobilization and therapy with blood products [9, 10]. Undoubtedly, she would have been a candidate for thrombosis chemoprophylaxis whether she had not had a susceptibility to life-threatening hemorrhage. Hypothetically, the massive retroperitoneal hematoma could have accelerated the clot formation process by compressing the inferior vena cava, slowing the venous backflow [11]. So that evacuation of the hematoma during the exploratory laparotomy might have ameliorated such a risk factor for preventing PE. Moreover, thrombosis chemoprophylaxis could have been started sooner if the bleeding source had been detected and ligated.

Some authors preferred conservative treatment for hemodynamically stable patients with massive peripartum retroperitoneal hematoma (especially in the pelvis), while their follow-up data for complications like subsequent thromboembolic events are limited [6, 12]. Starting coagulation prophylaxis should be delayed until ensuring a hematoma’s stability, which is time-consuming, and thus, risky for developing PE [12]. Taking such an adverse consequence into account is crucial when deciding to observe the patient rather than evacuating the hematoma and controlling the bleeding source.

Surgeons should consider that the ovarian and uterine arteries are established sources of hemorrhage in multiparous women presenting with unstable hemodynamics and retroperitoneal hematoma in their peripartum period [13]. By evacuating the hematoma and immediately ligating these arteries, such a patient may need no more invasive treatment, and experience less probability of thromboembolic complications. However, straightforward hematoma removal may exacerbate bleeding from other potential sources. With no current consensus on managing these patients, surgeons should weigh the costs and benefits of each potential care against each other.

Considering the same rationale for similar patients but with stable vital signs, TAE and percutaneous drainage of the hematoma may prevent the above consequences rather than conservation. After comprehensively searching the medical literature, Borghese *et al.* suggest a definite treatment for OAA in childbearing ages given its high risk of rupture [4]. Putting our experience and the evidence together, conservative treatment should be considered conservatively.

This report of a case cannot provide sufficient evidence to modify a standard protocol, but every patient should be managed on their individual basis. Sharing our experience with other clinicians may help them recognize knowledge gaps and plan the most appropriate management protocol while encountering such typical cases.

## CONCLUSION

Multiparous patients presenting with unstable hemodynamics and spontaneous retroperitoneal hematoma in their peripartum period are probably experiencing an ovarian or uterine artery aneurysm rupture. Regardless of the response to fluid or blood product administration or stability of the hematoma, surgeons may consider evacuating the hematoma and immediately ligating the ovarian and uterine arteries during exploratory laparotomies in such typical cases. This management may prevent further complications like the need for reoperation or PE.

## DATA AND MATERIAL AVAILABILITY

Available from the corresponding author upon reasonable request.
